# LncRNA CCAT1 functions as apoptosis inhibitor in podocytes via autophagy inhibition

**DOI:** 10.1002/jcb.29307

**Published:** 2019-08-29

**Authors:** Yanyan Su, Shuwen Yao, Shili Zhao, Jinchun Li, Hongyan Li

**Affiliations:** ^1^ Department of Nephrology, Huadu District People's Hospital of Guangzhou Southern Medical University Guangzhou China

**Keywords:** autophagy, lncRNA CCAT1, PI3K/Akt signaling, podocyte

## Abstract

Podocyte apoptosis importantly contributes to various kidney diseases. Long noncoding RNAs Colon cancer‐associated transcript‐1 (CCAT‐1) has been demonstrated for a critical role in cell proliferation. In the present study, the relationship between CCAT1 and popdocyte impairment, and the underlying mechanism was investigated. Podocytes were isolated from mice and then treated with tumor necrosis factor‐α to simulate podocyte injury. After developed CCAT1 overexpression or knockdown, cell viabilities were determined with the CCK‐8 assay, apoptosis was examined with Flow cytometry, the autophagy was observed by Western blot. Furthermore, phosphorylated PI3K and Akt expressions were examined. We found that after CCAT1 overexpression, the cell viability was significantly increased, apoptosis was significantly decreased, and autophagy was significantly inhibited, which was indicated by induced P62, LC3B‐I and decreased LC3B‐II. In addition, CCAT1 overexpression induced the levels of phosphorylated PI3K and Akt. With Rap treatment, these effects by CCAT1 were reversed. Furthermore, the results contrary to the effects by CCAT1 overexpression were presented after CCAT1 knockdown, and this was inhibited by 3‐MA. Taken together, our results suggested that CCAT1 induction critically participated in apoptosis inhibition in podocytes through autophagy inhibition via increasing PI3K/Akt signaling. This might act as a promising therapeutic intervention for renal diseases associated with podocyte apoptosis.

## 1 INTRODUCTION

Podocytes are visceral glomerular epithelial cells, lining outside of glomerular basement membrane and forming final glomerular filtration barriers to maintain the permeability of the GBM. Typically, podocyte injury results in marked proteinuria and associated with almost all glomerulopathies, including Focal segmental glomerulosclerosis (FSGS), membranous glomerulopathy, diabetes mellitus (DM), etc.^1^ FSFS and DM have been global health issues, and are leading causes of end‐stage renal disease (ESRD). It has been estimated that the number of DM patients will increase to 205 million in 2035 than in 2014, whereas FSGS recurred in approximately 40% of patients with transplantation, especially for high‐risk patients.^2^, ^3^ The diseases usually affect life quality and reduce graft survival. Therefore, identification of the pathways that protect podocyte from injury is essential for more effective therapies against glomerulosclerosis diseases.

Podocytes are terminally differentiated cells with high specification. They are lost after damage and cannot be replaced by new ones. To maintain homeostasis, autophagy is a critical mode to prevent cell injury.^4^, ^5^ Previous research have indicated that autophagy dysfunction was associated with podocyte injuries and massive proteinuria in patients with diabetes.^6^, ^7^ Autophagy is a highly conserved cellular process to maintain intracellular homeostasis by removing impaired protein or damaged organelles, and is essential for the survival of cells. Recent studies have suggested autophagy activation in podocytes may be a potential therapy to prevent the progression of nephropathy. Furthermore, it has been demonstrated that autophagy is renoprotective in many renal diseases, including acute kidney injury, obstructive nephropathy, and diabetic nephropathy.^8^

In many diverse glomerular diseases, podocytes are damaged by immune and nonimmune factors. Tumor necrosis factor (TNF)‐α, a pro‐inflammatory cytokine, plays an important role in some cellular responses, such as inflammation and cell death.^9^ A recent has shown that TNF‐α induce podocyte apoptosis.^10^ On the other hand, inhibition of the TNF‐α activity presents protective effects on glomerulosclerosis and on podocyte apoptosis.^11^

Long noncoding RNAs (lncRNAs) are RNA molecules generally composed of more than 200 nucleotides in length without protein‐coding capacity, and involved in various important cellular processes, such as cell proliferation, apoptosis, differentiation, and tumor cell invasion.^12^, ^13^ Colon cancer‐associated transcript‐1 (CCAT1) is a type of lncRNA that is closely associated with a variety of cancers.^13^, ^14^ Furthermore, a few studies have reported that CCAT1 importantly contributes to the regulation of cell apoptosis and autophagy.^13^

In this study, it was observed that CCAT1 expression level in podocytes was significantly decreased after TNF‐α stimulation. Then, we evaluated the role of CCAT1 in the viability of podocytes. These results suggest the novel regulatory function of CCAT1 in TNF‐α induced podocyte injury and provide a potential treatment of glomerulosclerosis diseases.

## 2 MATERIALS AND METHODS

### 2.1 Podocyte isolation and culture

The male mice used for cell isolation were 6 to 8 weeks old and obtained from the Laboratory Animal Center of Southern Medical University. All the performances on the mice were approved by the Animal Care and Use Committee of Southern Medical University. Podocytes were isolated as previously reported.^10^ In brief, the kidneys were collected from the anesthetized mice, minced, and gently ground. The resulting suspension was filtered through meshes and centrifuged. The pellet was incubated in RPMI 1640 (Procell, Wuhan, China) with 0.1% collagenase I. After centrifugation at 100 g for 5 minutes, the primary podocytes were maintained in complete medium on polylysine‐coated dishes with at 37°C, 5% CO_2_ for a subsequent test.

### 2.2 Cell transfection and treatments

The primary precursor sequence and the specific shRNA of CCAT1 were obtained from GenePharma Company (Shanghai, China), and then cloned into pLV‐GFP vector (Addgene, MA) for overexpression and pGLVU6/GFP vector for knockdown, respectively, with Lentiviral Packaging Mix (Genewiz, Suzhou, China). The transfected cells were selected with G418. Concurrently, the cells transfected with the empty vector were used as negative control (NC). In the tests with podocyte cells transfected with CCAT1 overexpression or knockdown, a group treated with rapamycin (Rap, 2.5 μM) or 3‐methyladenine (3‐MA, 5 mM; Sigma, MO) before TNF‐α exposure was included.

### 2.3 Cell viability and proliferation assay

With 96‐well plates, podocytes were seeded at 10^4^ cells/well in 100 μL complete medium. After culture overnight at 37°C with 5% CO_2_, the cells were exposed to control or TNF‐α at 5 ng/mL for 24 hours, 48 hours, or 72 hours. CCK‐8 (10 μL; Beyotime, Shanghai, China) solution was added into each well and then incubated for another 1 hour. Absorbance was examined at 450 nm by a microplate reader (Bio‐Rad, Hercules, CA).

With BD FACSCalibur flow cytometer (BD Biosciences, CA, USA), cell proliferation was determined by 5‐ethynyl‐20‐deoxyuridine (EdU) incorporation. According to the protocol of EdU Apollo 488 In Vitro Flow Cytometry Kit (RiboBio, Guangzhou, China), EdU of 20 μL was added into each well and the cells received another 2 hours incubation. After collection and centrifugation, the cells were fixed with 4% paraformaldehyde for 30 minutes, stained with anti‐EdU working solution for 30 minutes. Finally, EdU‐positive cells were quantified using flow cytometry,

### 2.4 Cell apoptosis

After incubated with 5 ng/mL TNF‐α for 48 hours, the podocytes were collected with 0.25% ethylenediamine tetraacetic acid (EDTA)‐free trypsin. After centrifugation at 1000 rpm for 5 minutes, the cell pellet was re‐suspended in pre‐cooled PBS, fixed overnight in pre‐cold 70% ethanol at 4°C and then incubated in a binding buffer with Annexin V‐FITC and propidium iodide (PI; KeyGen, Nanjing, China) for 15 minutes in the dark. Finally, the stained cells were examined by BD FACSCalibur flow cytometer (BD Biosciences).

### 2.5 Quantitative real‐time reverse transcription PCR

According to the manufacturer's instructions, total RNA was isolated from podocytes with TRIzol (Invitrogen, MA) and was reversed into cDNA by PrimeScript RT‐PCR kits (TaKaRa, Dalian, China). By SYBR Green PCR kits (Roche Penzberg, Upper Bavaria, Germany), quantitative real‐time reverse transcription PCR (qRT‐PCR) was implemented using ABI 7500 Real‐time PCR System (Appied Biosystems). The primers were as follows: CCAT1, forward: 5′‐TCACTGACAACATCGATTTGAAG‐3′, reverse: 5′‐GGAG AAAACGCTTAGCCATACAG‐3′; GAPDH, forward 5′‐ACTTTGTCAAGCTCATTTCC‐3′, reverse 5′‐TGCAGCGAACTTTATTGATG‐3′. GAPDH was used as the internal control and fold change of CCAT1 was calculated using $2^{-▵▵C_{t}}$.

### 2.6 Western blots

The cells were collected in RIPA buffer containing protease inhibitor cocktail (Beyotime, Haimen, China). Proteins were determined using the BCA Protein Assay (Beyotime) and denatured by heat. Equal amounts (20 μg) of protein in loading buffer were added into each well of the SDS‐PAGE gel, separated by electrophoresis, transferred onto PVDF membranes. After blocking by 5% skim milk in TBST (TBS + Tween 20) for about 1 hour at room temperature, the membranes were incubated with primary antibodies against P62 (catalog no. #5114), LC3B (catalog #2775), Bax (catalog #5023), Bcl‐2 (catalog #3498), p‐PI3K (catalog #4228), p‐Akt (catalog #4085), and GAPDH (catalog #5174) at 4°C overnight. All the antibodies were obtained from Cell Signaling (MA). After washed three times with TBST, the membranes were incubated with secondary antibody conjugated with horseradish peroxidase (catalog no. sc‐2030; Santa Cruz, CA) for 1 hour at room temperature. Finally, the immunoreaction was detected with an ECL detection kit (Amersham, GE Healthcare).

### 2.7 Statistical analysis

The data were presented as the mean ± SD of three independent tests. Two‐tailed Student *t* tests or one‐way ANOVA was used for statistical analysis with SPSS 21.0 (SPSS Inc, Chicago, IL) software, and *P* < .05 was considered statistical differences.

## 3 RESULTS

### 3.1 TNF‐α decreased CCAT1 expression and autophagy

**Figure 1:**
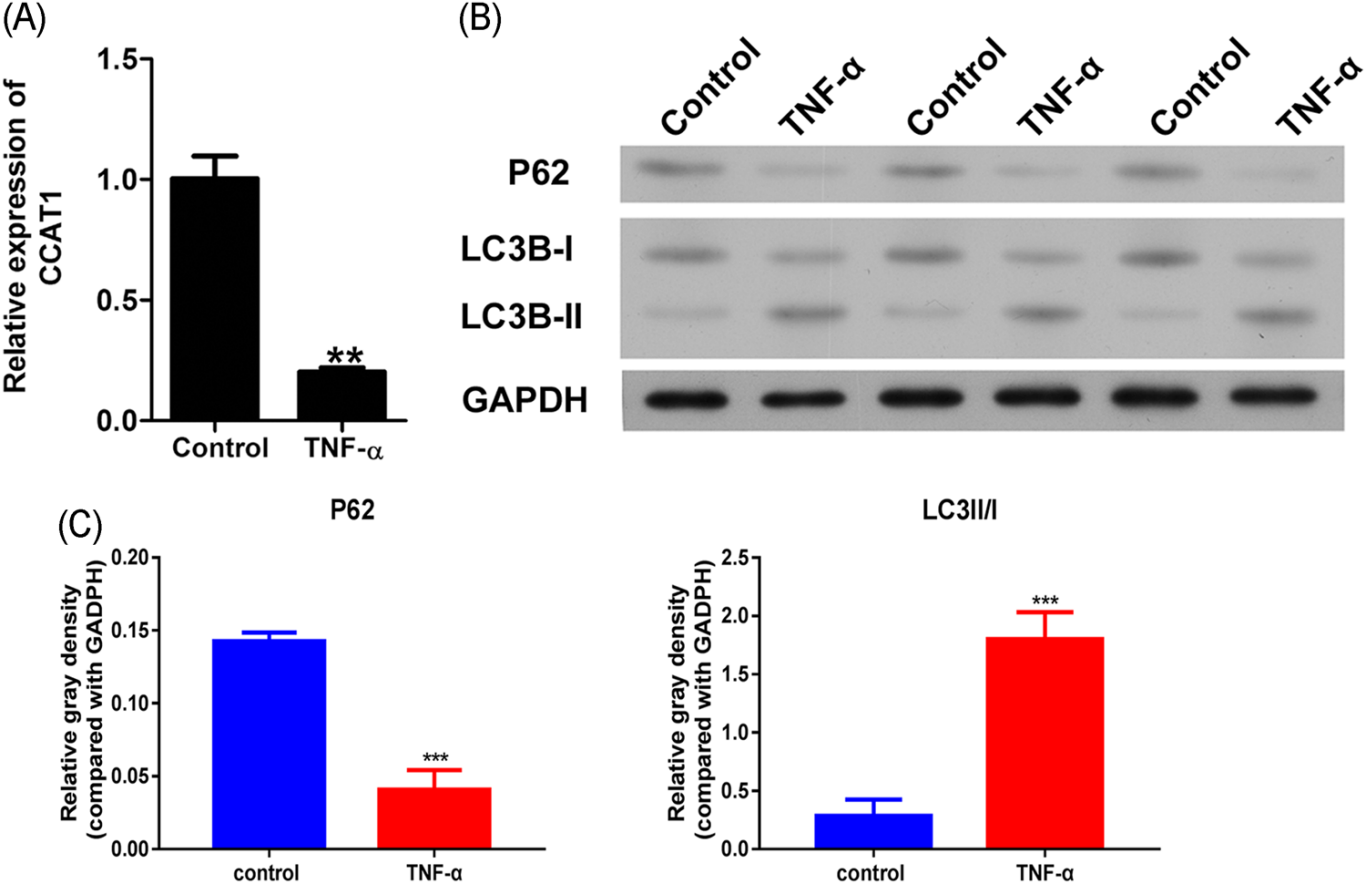
Effects of TNF‐α on CCAT1 expression and autophagy in podocytes. Podocytes were exposed to TNF‐α for 48 hours, then CCAT1 was determined by qRT‐PCR (A), and the expressions of autophagic markers, P62 and LC3B, were determined by Western blot (B), quantified and normalized to GAPDH using the ImageJ software (C). CCAT1, colon cancer‐associated transcript‐1. **P <* *.01, ***P <* *.001, compared with the control group. CCAT1, colon cancer associated transcript‐1

Firstly, CCAT1 expression was determined in podocyte injury model established by exposure to 5 ng/mL TNF‐α for 24 hours. As shown in Figure 1A, it was observed that CCAT1 expression was significantly decreased in the podocyte injury model as compared with the control, indicating that CCAT1 might protect podocyte from injury. Subsequently, LC3B and P62 were determined to observe the change of autophagy in the cell injury model. As shown in Figure 1B,C, P62 expressions were markedly decreased after TNF‐α treatment. Similarly, significantly decreased LC3BI was observed in podocyte injury model, whereas LC3BII significantly increased. The changes in the autophagy marker indicated that autophagy was significantly decreased in the injury model.

### 3.2 Effect of CCAT1 overexpression on podocyte viability and proliferation

**Figure 2:**
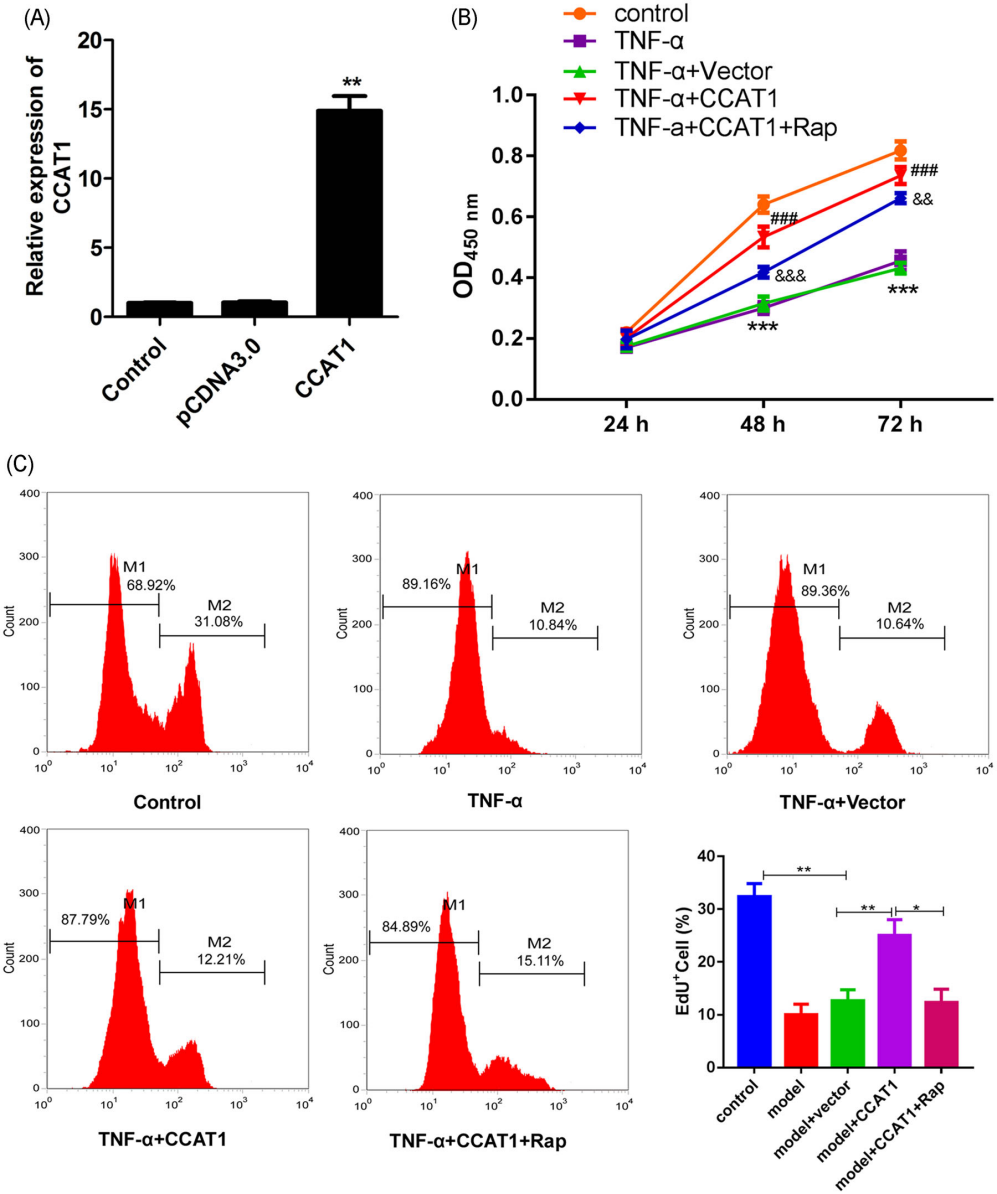
Effects of CCAT1 overexpression on podocyte viability In the parent podocytes or the podocytes transfected with empty vector or CCAT1, qRT‐PCR assay was performed to examine the expression of CCAT1 (A), **P <* *.01, compared with the pcDNA3.0 group. The cell viabilities were determined with the CCK‐8 assay after exposure to control, TNF‐α alone for 24 hours, 48 hours, or 72 hours, or in combination with Rap (B). ***P <* *.001, compared with the control group; ^###^ *P* < .001, compared with the group of TNF‐α + vector; ^&&^ *P* < .01, ^&&&^ *P* < .001, compared with model + CCAT1. At 48 hours after treatments, the cell proliferation was determined the Edu assay. The EdU stained positive cells were represented by M2 and considered in proliferation (C). CCAT1, colon cancer associated transcript‐1; EdU, 5‐ethynyl‐20‐deoxyuridine; Rap, rapamycin. *P < .05, **P < .01

The CCK‐8 assay was used to examine the effect of CCAT1 overexpression on the viability of podocytes exposed to TNF‐α. At first, we confirmed the overexpression of CCAT1, which was supported by the significant increase when compared with the controls (Figure 2A). The viabilities assay of parent podocytes and those transfected with empty vector showed that the cell viabilities and proliferation were significantly decreased after TNF‐α treatment for 48 hours and 72 hours, indicating the cell injury model was successfully established (Figure 2B). As expected, the decrease in the cell viability was significantly suppressed after CCAT1 overexpression, and this suppression was significantly inhibited after treatment with Rap, an inducer of autophagy. In the proliferation assay, consistent data was observed (Figure 2C). These data indicated that CCAT1 played an important role in protecting podocytes from injury by TNF‐α and this might be through modulating autophagy. Virus‐based gene delivery system was also used to detect the effect of CCAT1 overexpression on podocyte viability and proliferation. As shown in Figure S1, the transfection efficiency was first detected by qRT‐PCR (Figure S1A). In line with the previous result, overexpression of CCAT1 significantly reversed the decreased viability and proliferation of podocyte exposed to TNF‐α, and this reverse was inhibited after Rap treatment (Figure S1B,S1C).

### 3.3 Effect of CCAT1 overexpression on podocyte apoptosis and autophagy

**Figure 3:**
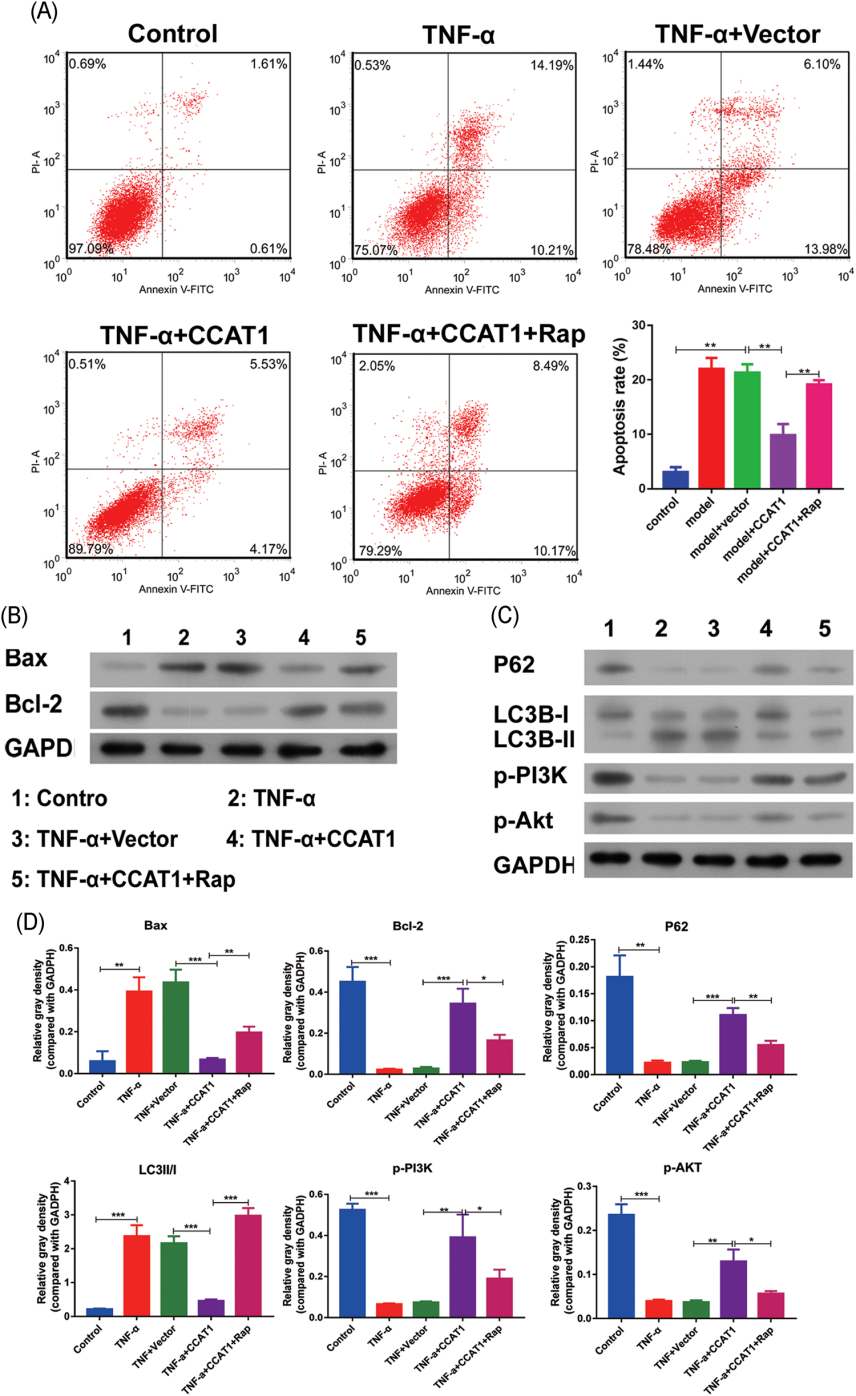
Effects of CCAT1 overexpression on podocyte apoptosis and autophagy The podocytes transfected with CCAT1 or with the empty vector were exposed to TNF‐α for 48 hours or in combination with Rap. Meanwhile, the parent podocytes were treated with TNF‐α alone or with vehicle as control. After treatments, the cell apoptosis was determined by Flow cytometry (A). By Western Blot, the proteins associated with cell apoptosis, Bcl‐2 and Bax, were examined (B), those associated with autophagy, P‐62 and LC3B, as well as p‐PI3K and p‐Akt was determined (C). The band intensities were quantified and normalized against GAPDH (D). CCAT1, colon cancer associated transcript‐1; EdU, 5‐ethynyl‐20‐deoxyuridine; Rap, rapamycin. *P < .05, **P < .01, ***P < .001

Flow cytometry was used to examine the apoptosis of podocytes. It was observed that apoptosis was significantly increased in the cell injury model, and this was markedly suppressed in the cells with CCAT1 overexpression (Figure 3A). On the other hand, the apoptosis of CCAT1 overexpressing cells was markedly increased after Rap treatment. The examination of pro‐ and antiapoptotic proteins showed the consistent results that Bax and Bcl‐2 were, respectively, significantly increased and decreased in the injury cell model, and the alterations were reversed in the cells with CAAT1 overexpression (Figure 3B,D). In addition, the reversal effect was significantly inhibited after exposure to Rap. All these indicated that CCAT1 importantly contribute to podocyte survival and this was inhibited by Rap.

Subsequently, we observed the autophagy of podocytes. As shown in Figure 3C,D, P62, LC3B‐I was significantly increased in cells overexpressing CCAT1 as compared with the cell injury model, whereas LC3B‐II was significantly decreased. Furthermore, the inductive and inhibitory effects above mentioned were significantly inhibited after Rap treatment. These indicated that CCAT1 played an important inhibitory role in the autophagy of podocytes induced by TNF‐α, and this effect can be suppressed by Rap. A further investigation showed that phosphorylation of PI3K and Akt was markedly increased by CCAT1 overexpression as compared with the cell injury model. However, the inductive effect was significantly inhibited after Rap treatment. All these results indicated that CCAT1 overexpression might inhibit cell apoptosis via inhibition of autophagy, and induced phosphorylation of PI3K and Akt might play an important role.

### 3.4 Effect of CCAT1 inhibition on podocyte viability, apoptosis, and autophagy

**Figure 4:**
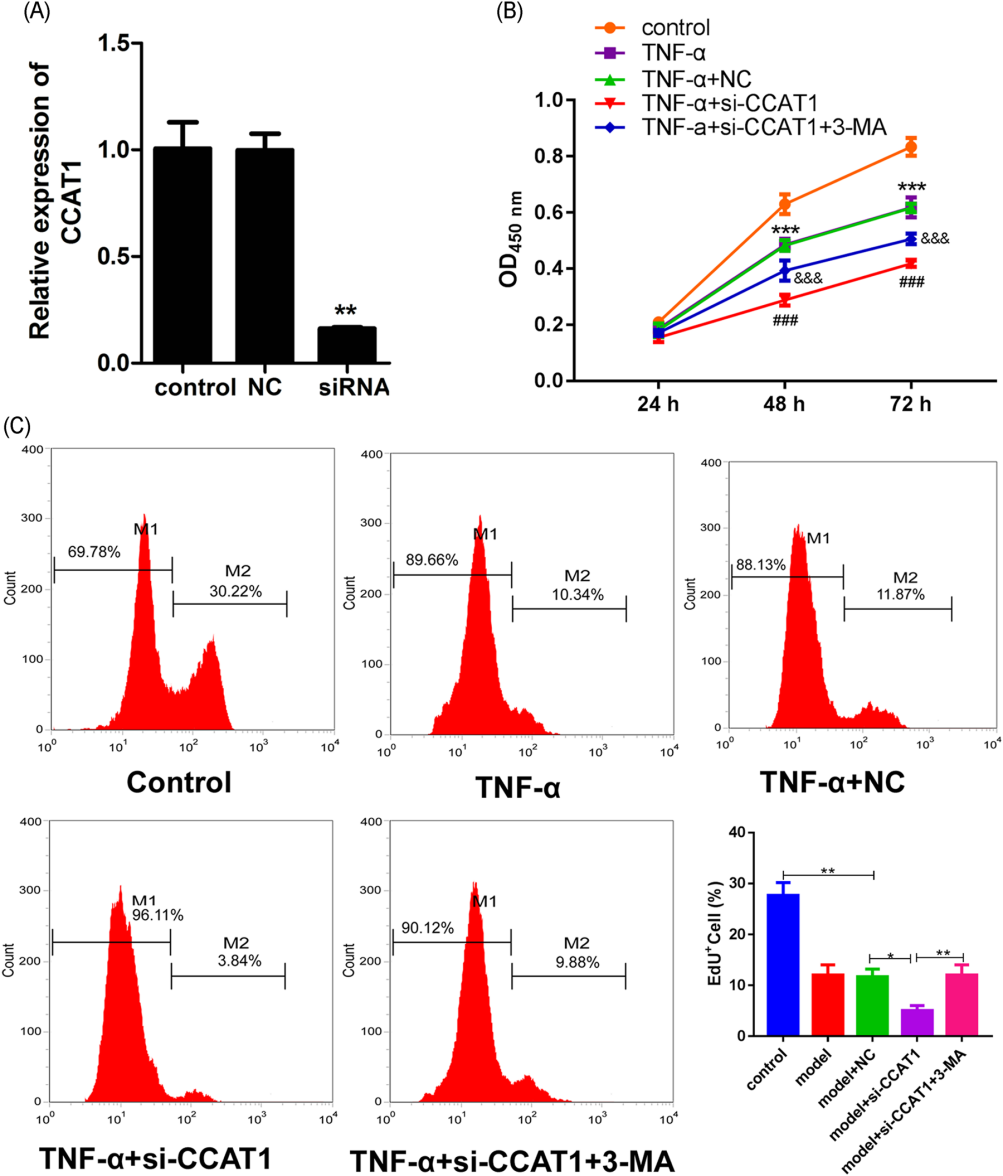
Effects of CCAT1 knockdown on podocyte viability The siRNA against CCAT1 (si‐CCAT1) or the negative control (NC) was transfected into the podocytes. Subsequently, the qRT‐PCR assay was performed to examine the expression of CCAT1 in the transfected and the parent cells (A). **P <* *.01, compared with the NC group. The parent and transfected cells were treated with TNF‐α for 24 hours, 48 hours, or 72 hours alone, or in combination with 3‐MA. After treatments, the cell viabilities were determined with the CCK‐8 assay (B), ***P <* *.001, compared with the control group; ^###^ *P* < .001, compared with the group of TNF‐α + NC; ^&&&^ *P* < .001, compared with TNF‐a + si‐CCAT1. After treatments for 48 hours, the cell proliferation was determined using flow cytometry after the EdU assay (C). CCAT1, colon cancer‐associated transcript‐1; EdU, 5‐ethynyl‐20‐deoxyuridine. **P < .01, compared with the control group; ^##^ *P* < .01, compared with the group of TNF‐α + NC, ^#^ *P* < .05, compared with the group of TNF‐α + si‐CCAT1. 3‐MA, 3‐methyladenine. *P < .05, **P < .01

To furthermore confirm the effect of CCAT1 on podocyte injury, CCAT1 knockdown was performed. As shown in Figure 4A, CCAT1 expression was significantly inhibited after shRNA transfection, indicating the knockdown cells were successfully established. Subsequently, it was observed that CCAT1 knockdown further decreased the viability and proliferation of podocytes when treated with TNF‐α (Figure 4B,C). The inhibitory effect induced by CCAT1 knockdown was significantly suppressed after 3‐MA treatment, an inhibitor of autophagy. These indicated that CCAT1 inhibition significantly contributed to podocyte injury, and this might be through induction of autophagy. Virus‐based gene delivery system was also used to detect the effect of CCAT1 knockdown on podocyte viability and proliferation. As shown in Figure S2, the transfection efficiency was firstly detected by qRT‐PCR (Figure S2A). In line with the previous result, knockdown of CCAT1 decreased podocyte viability and proliferation as compared with the injury model (Figure S2B,S2C). After 3‐MA treatment, the increased effect was significantly suppressed (Figure S2B,S2C).

### 3.5 Effect of CCAT1 inhibition on podocyte apoptosis and autophagy

**Figure 5:**
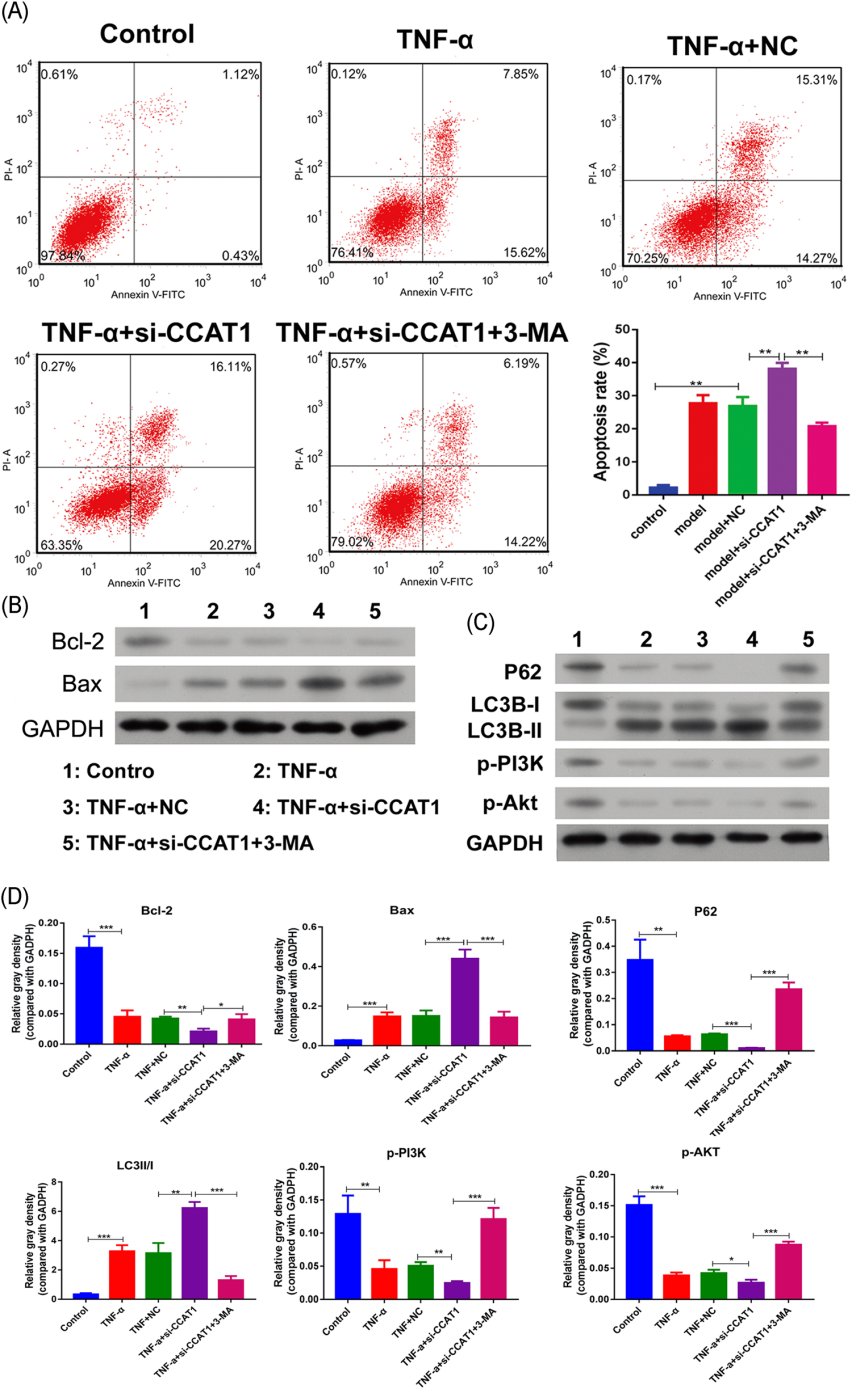
Effects of CCAT1 knockdown on podocyte apoptosis and autophagy The parent podocytes and those transfected with siRNA against CCAT1 (si‐CCAT1) or the negative control (NC) were exposed to TNF‐α for 48 hours, or in combination with 3‐MA. The cell apoptosis was determined by Flow cytometry (A). By Western Blot, the proteins associated with cell apoptosis, Bcl‐2 and Bax, were examined (B), and those associated with autophagy, P‐62 and LC3B, as well as p‐PI3K and p‐Akt was determined (C). The expressions were quantified by software of ImageJ and normalized to GAPDH (D). 3‐MA, 3‐methyladenine; CCAT1, colon cancer associated transcript‐1. *P < .05, **P < .01, ***p < .001

Contrary to CCAT1 overexpression, the cell apoptosis was significantly induced after CCAT1 knockdown when compared with the injury model, and this was significantly inhibited by 3‐MA treatment (Figure 5A). Consistently, it was observed that Bcl‐2 and Bax expressions were obviously inhibited and increased, respectively, in the cells with CCAT1 knockdown, and the effects were significantly suppressed by 3‐MA treatment (Figure 5B,D). These data indicated that CCAT1 inhibition might play an important role in podocyte apoptosis.

The autophagy marker determination showed that CCAT1 knockdown significantly inhibited P62 and LC3B‐I expressions, whereas increased LC3B‐II expression (Figure 5C,D). The results indicated that CCAT1 inhibition might significantly increase podocyte autophagy, which might importantly contribute to the cell apoptosis. PI3K signaling examination showed that phosphorylated PI3K and Akt were significantly inhibited after CCAT1 knockdown (Figure 5C,D). Consistent with the effect on cell apoptosis and autophagy, 3‐MA treatment significantly inhibited the inhibitory effect on p‐PI3K and p‐Akt by CCAT1 knockdown (Figure 5C,D). These indicated that the effects of CCAT1 inhibition on cell apoptosis and autophagy might be achieved by inhibiting PI3K/Akt signaling.

## 4 DISCUSSION

Podocyte injury or apoptosis has been suggested to initiate various glomerulosclerosis diseases.^2^, ^15^ lncRNAs, such as CCAT1, can participate in the regulation of cell proliferation, migration, and apoptosis by controlling the downstream pathways.^13^ Therefore, we studied the role of CCAT1 in podocyte injury induced by TNF‐α and investigate the underlying mechanism. In the present study, it was firstly reported that CCAT1 inhibition critically contributes to TNF‐α induced podocyte apoptosis and this might be associated with the PI3K/Akt signaling pathway.

CCAT1 is a widely accepted oncogenic lncRNA. Emerging studies have suggested that CCAT1 is upregulated in a great many types of cancers, and critically contributes to cell proliferation.^12^, ^16^ It has been demonstrated to be regulated by some factors. In gastric, colon and live cancers, c‐MYC could enhance CCAT1 expression by directly binding to the promoter region of the IncRNA.^17^, ^18^ Jiang et al^17^ reported that TP63 and SOX2 also can bind to the promoter in squamous cell cancers, resulting in induced transcription activity. Moreover, it has been suggested that regulation of CCAT1 expression might be tissue‐specific.^17^ To the best of our knowledge, there is no report on the correlation between TNF‐α and CCAT1. In this study, we observed TNF‐α exposure significantly decreased CCAT1 expression in podocytes.

lncRNAs have been observed to interact with chromatin, associated with transcriptional regulation, and RNA processing by binding to DNA, RNA, and transcription factors. CCAT1 was detected to be present both in nucleus and cytoplasm,^17^, ^19^ indicating it can additionally modulate stability or translation of transcripts.^20^, ^21^, ^22^ A previous study has noted that CCAT1 can interact with a transcription factor to increase the activity of MYC promoter and its enhancers, leading to increased expressions of MYC.^23^ Likely, CCAT1 regulates EGFR expressions in SCC cells.^17^ By enhancing the stability of targeting protein, CCAT1 upregulates the expression level of the protein, such as Livin, a protein generally regarded as an apoptosis inhibitor in various malignancie.^24^ As a fundamental function by IncRNA, CCAT1 can regulate expressions of many miRNAs, including miR‐143, miR‐155, miR‐181a, and miR‐218‐5p.^12^, ^25^, ^26^ All these indicate that CAAT1 is a master regulator, and can resultantly affect a variety of cellular signaling pathways, including Wnt/β‐catenin, MEK/ERK1/2, and PI3K/AKT signaling pathways.^13^, ^17^, ^25^

Previous studies have shown that PI3K/Akt is widespread in cells, and is vital for the regulation of cell proliferation, differentiation, apoptosis, and metabolism.^5^, ^27^ The signaling pathway activation plays a critical role in podocyte cytoskeleton structure stabilization and podocyte apoptosis inhibition.^28^ In the present study, it was observed that CAAT1 inhibition resulted in PI3K/Akt inhibition. It has been shown that the PI3K/Akt pathway can be regulated by the targets of CCAT1, including miR‐143 and EGFR.^17^, ^25^ Therefore, inhibition of CCAT1 in this study might regulate the activation of PI3K/Akt activation by either one of them, but this needs further investigation.

Autophagy dysfunction has been demonstrated to be associated with podocyte injuries. In addition to Atg5, Atg7, and LC3, mTOR is a classic regulator of autophagy activation in podocytes.^29^, ^30^ On the basis of the critical role of PI3K/Akt in mTOR activation, PI3K/Akt critically regulate cell autophagy. In this study, it was observed that PI3K/Akt inactivation increased autophagy. Consistently, it has been reported that the activated Akt‐mTOR signaling pathway suppresses autophagy, and suppressing the mTOR activity induces autophagy.^31^, ^32^ On the contrary, it has also been reported that PI3K/Akt activation induces autophagy in podocytes and plays a protective role.^33^ The variation might be because of the different type of stimuli. Autophagy in podocytes plays a detrimental role in apoptosis. Autophagy and apoptosis share several critical proteins and as a result, the activation of autophagy may increase the activities of these proteins, and eventually promote apoptosis.

In conclusion, our results confirmed that CCAT1 may be involved in podocyte autophagy and apoptosis. This study also revealed that CCAT1 inhibition induced podocyte apoptosis by inhibiting the PI3K/Akt pathway. Accordingly, CCAT1 might be a novel therapeutic target for diseases associated with pododcyte injury.

## CONFLICT OF INTEREST

The authors declare that there is no conflict of interest.

## DATA AVAILABILITY

The data used in this research are available form the corresponding author on reasonable request.

## Supporting information

Supporting information

Supporting information

Supporting information
