# Supplementary figures and images for: LncRNA CCAT1 functions as apoptosis inhibitor in podocytes via autophagy inhibition
Source: J Cell Biochem. 2019 Aug 29;121(1):621–31. doi: 10.1002/jcb.29307 (PMC6899777; doi:10.1002/jcb.29307)

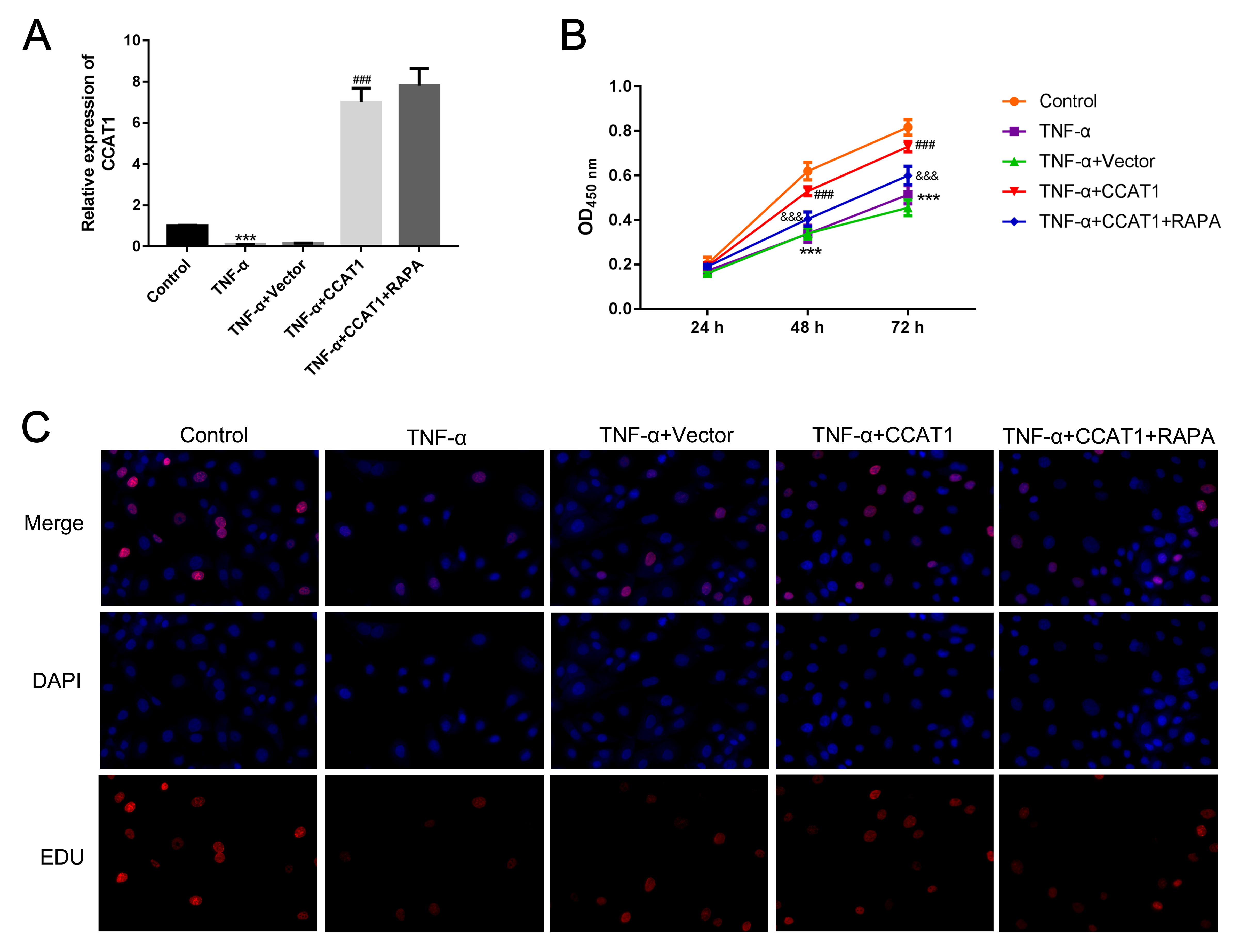

Supplement: Supplementary file 1 — Supporting information [file JCB-121-621-s001.tif]

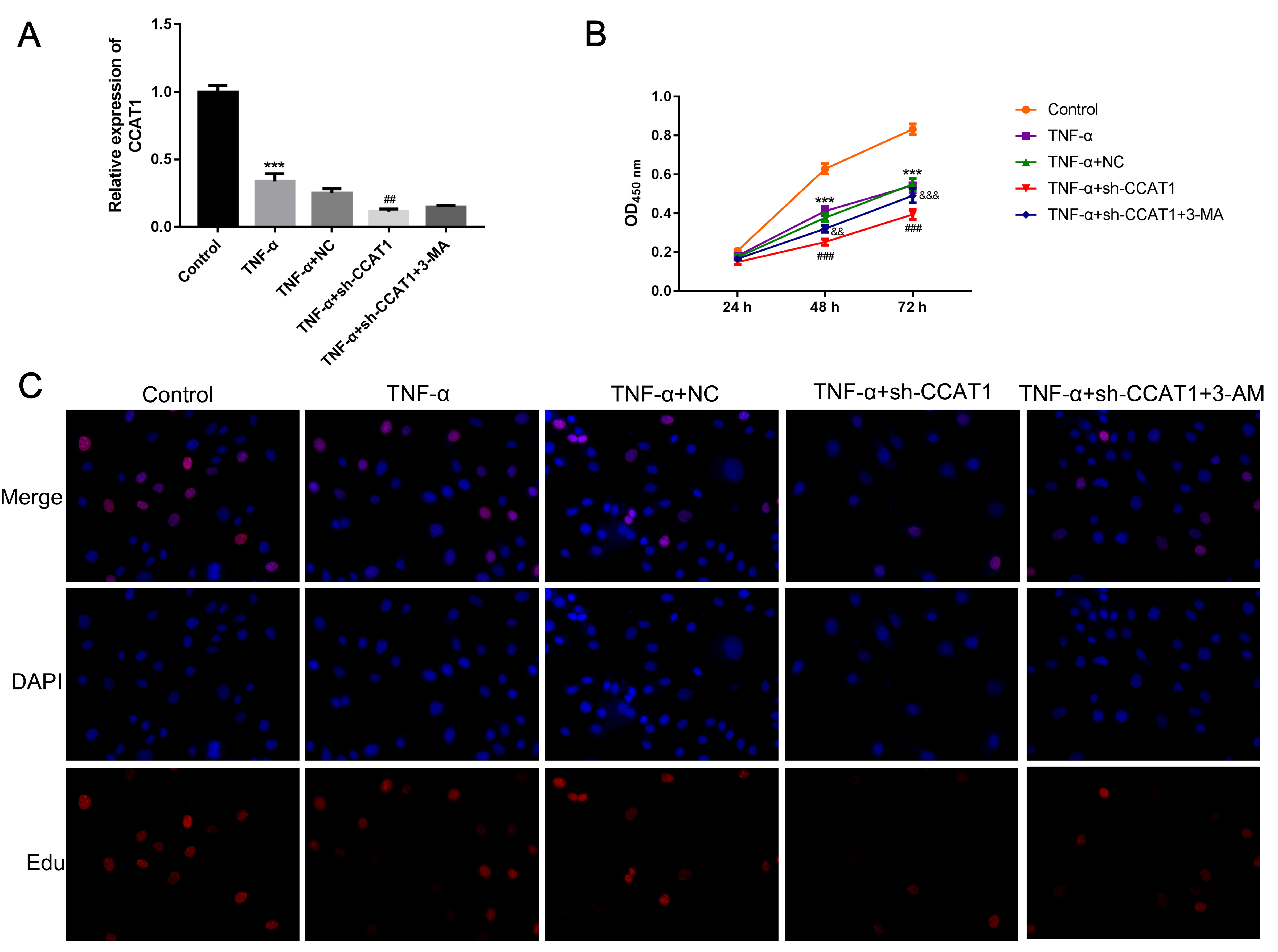

Supplement: Supplementary file 2 — Supporting information [file JCB-121-621-s002.tif]
