# Supplementary material for: LncRNA CCAT1 functions as apoptosis inhibitor in podocytes via autophagy inhibition
Source: J Cell Biochem. 2019 Aug 29;121(1):621–31. doi: 10.1002/jcb.29307 (PMC6899777; doi:10.1002/jcb.29307)
Supplement: Supplementary file 3 — Supporting information [file JCB-121-621-s003.docx]

**Supplementary Figure S1. Effect of CCAT1 overexpression on podocyte viability and proliferation**

CCAT1 in pLV-GFP vector or the empty vector as negative control (NC) was transfected into podocytes. The parent and transfected cells were treated with TNF-α for 24h, 48h or 72h alone, or in combination with Rap. After treatments, the expression of CCAT1 was determined by qRT-PCR in the transfected and the parent cells (A), the cell viabilities were determined with CCK-8 assay (B), and the cell proliferation was examined using EdU staining (C). ****P<*0.001, compared with the control group; ^###^*P*<0.001, compared with the group of TNF-α+vector; ^&&&^*P*<0.001, compared with TNF-a+CCAT1. Rap, rapamycin.

**Supplementary Figure S2. Effect of CCAT1 knockdown on podocyte viability and proliferation**

ShRNA against CCAT1 (sh-CCAT1) in pGLVU6/GFP vector or the empty vector as negative control (NC) was transfected into podocytes. The parent and transfected cells were treated with TNF-α for 24h, 48h or 72h alone, or in combination with Rap. After treatments, the expression of CCAT1 was determined by qRT-PCR in the transfected and the parent cells (A), the cell viabilities were determined with CCK-8 assay (B). The cell proliferation was examined using EdU staining after 48-hour treatment (C). ****P<*0.001, compared with the control group; ^##^*P*<0.01, ^###^*P*<0.001, compared with the group of TNF-α+NC; ^&&^*P*<0.01, ^&&&^*P*<0.001, compared with TNF-a+sh-CCAT1. 3-MA, 3-methyladenine.
